# Differentiation of severe bilateral panuveitis following phacoemulsification: a case report

**DOI:** 10.1186/s12886-016-0252-y

**Published:** 2016-06-08

**Authors:** Xi Peng, Junjun Zhang

**Affiliations:** Department of Ophthalmology, West China Hospital, Sichuan University, No.37 Guoxue Xiang, Chengdu, 610041 Sichuan China

**Keywords:** Behcet’s disease, Endophthalmitis, Phacoemulsification, Cataract surgery, Late complication

## Abstract

**Background:**

Cataract surgery typically offers instant visual rehabilitation with rare postoperative complications. However, if complications occur, these complications may be confusing and threatening. We present a case of severe bilateral panuveitis following phacoemulsification and intraocular lens implantation and discuss the importance of a correct diagnosis and management.

**Case presentation:**

A 75-year-old Asian male with bilateral phacoemulsification and intraocular lens implantation developed severe inflammation with sharp vision loss in both eyes after the surgeries. Physical examination indicated bilateral panuveitis. With a presumptive diagnosis of suppurative endophthalmitis and a history of effective treatment with intravenous antibiotics plus ofloxacin and steroid drops, intravenous ceftazidime and vancomycin were administered. However, the effects were minimal. With a supplemental history of recurrent oral, perineal, and gastrointestinal ulcers, a diagnosis of Behcet’s disease was made, and systemic immune inhibitors were prescribed instead of invasive treatments, which might exacerbate the condition. After 5 days of medication, the inflammation was markedly relieved, and no recurrence was observed 2 weeks later.

**Conclusion:**

Correct differentiation of confusing conditions is crucial to implement appropriate management. Postoperative complications of cataract surgery should be differentiated carefully, and perioperative management in patients with autoimmune uveitis should be provided with caution.

## Background

Cataract surgery typically obtains instant visual rehabilitation and is extensively performed today. Given the surgery’s effectiveness and high safety profile, both patients and ophthalmic surgeons have high expectations for the outcome. Thus, serious postoperative complications threatening visual acuity have attracted considerable attention from both surgeons and patients. Correct differentiation of confusing conditions is crucial to implement the appropriate management. We present a case of serious bilateral panuveitis following uncomplicated phacoemulsification and intraocular lens (IOL) implantation in an elderly male and discuss the importance of a correct diagnosis and management.

## Case presentation

**Fig. 1:**
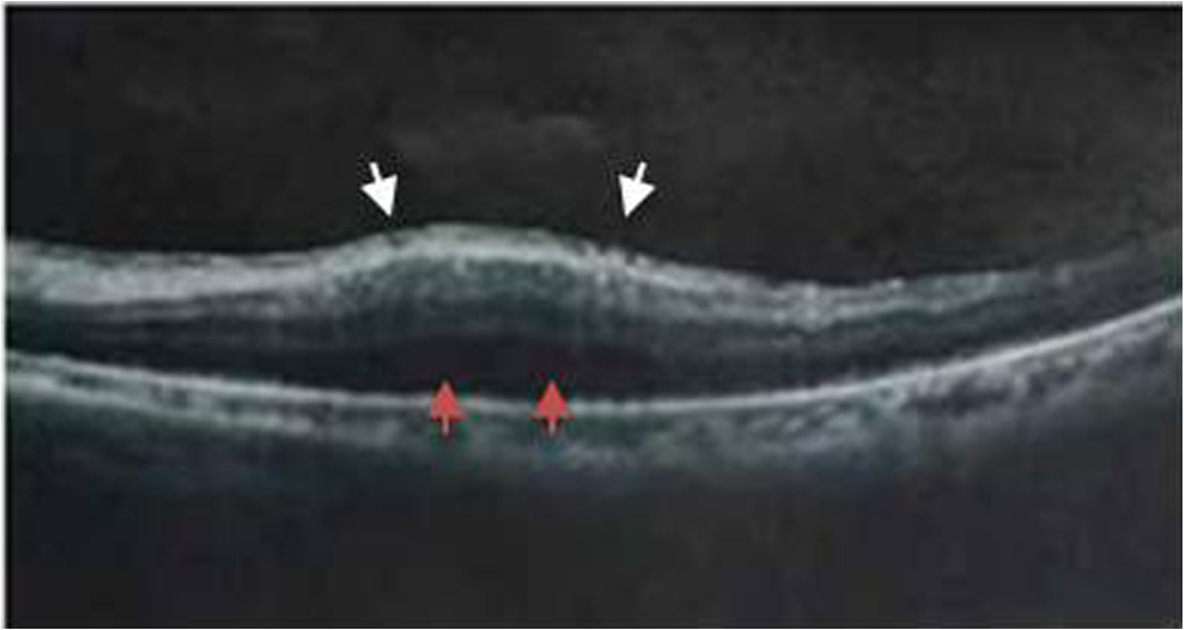
Optical coherence tomography (OCT) demonstrated epimacular membrane (*white arrows*) and local serous neuroepithelial detachment (*red arrows*) in the left eye

**Fig. 2:**
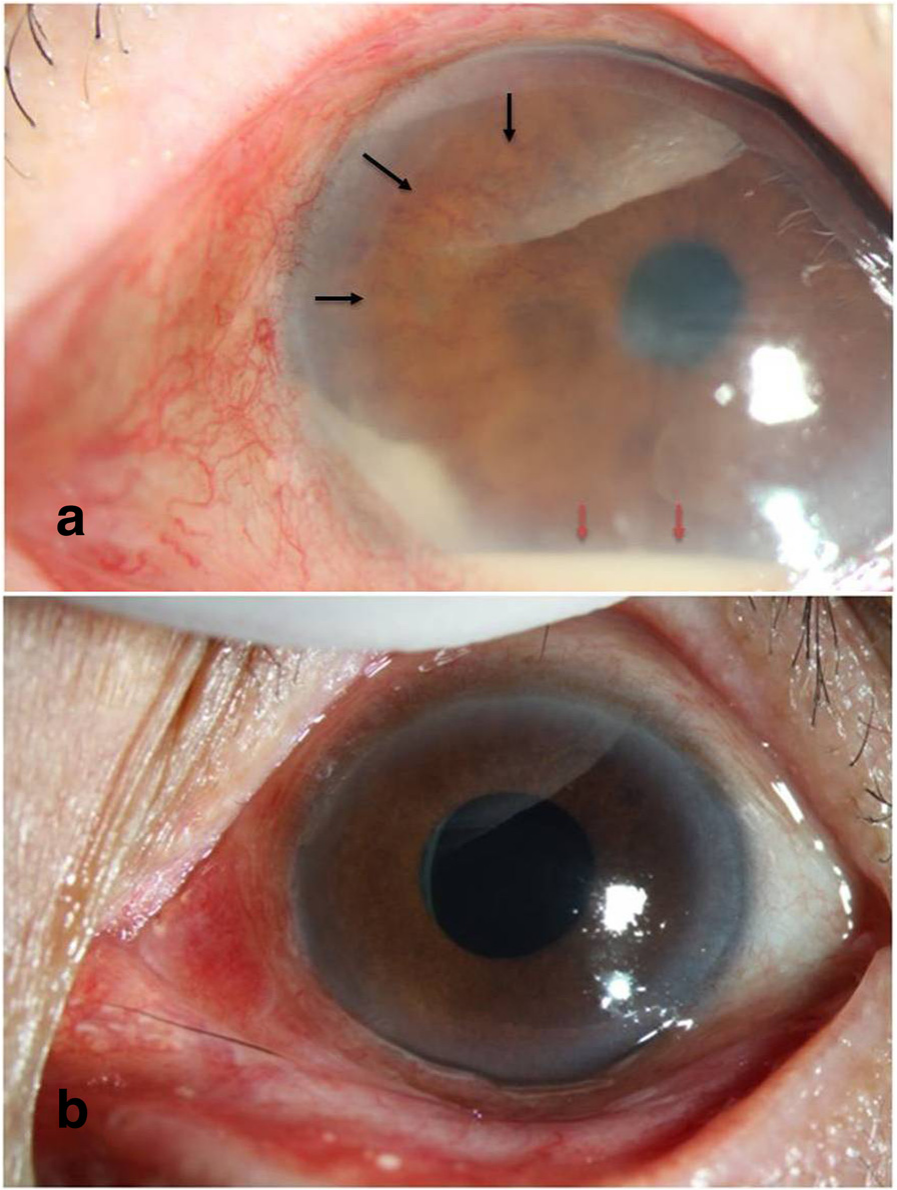
**a** Prior to immunosuppressive therapy, the left eye had deep ocular redness, corneal edema, 2-mm hypopyon (*red arrows*) and iris neovascularization (*black arrows*). However, the hypopyon was relatively quiet with few cells and little aqueous flare. **b** After immunosupressive therapy, the anterior chamber reaction was obviously reduced in the left eye, and the hypopyon was completely absorbed 5 days after oral prednisone, thalidomide and cyclophosphamide were added

A 75-year-old man was admitted to our hospital emergently with a presumptive diagnosis of suppurative endophthalmitis after bilateral cataract surgery in another hospital. The perioperative history of the cataract surgery was reviewed. This patient had bilateral blurred vision for 2 years. No anterior inflammation was reported preoperatively. The fundus “could not be observed by ophthalmoscopy.” Optical coherence tomography (OCT) revealed epimacular membrane and local serous neuroepithelial detachment in the left eye (Fig. 1); ultrasound revealed bilateral vitreous opacity. After uneventful phacoemulsification and IOL implantation in both eyes, visual acuity (VA) values were 0.2 (right) and 0.3 (left). No keratic precipitates (KP) or aqueous flare was noted, but mild opacity in vitreous was observed. Postoperative antibiotics and steroid drops were prescribed for 1 month. Five weeks after the first surgery (the right eye), he suffered sharp vision loss, hand motion (HM), in the right eye with severe panuveitis and was diagnosed with endophthalmitis. After a week of intravenous levofloxacin and cefazolin plus ofloxacin and steroid drops, the VA recovered to 0.15 with little anterior chamber reaction. Two months later, the left eye underwent a similar pattern. Again, the condition was diagnosed and treated in a similar manner. Remission was obtained 1 week later. After recurrent inflammatory episodes in both eyes over a 6-month period, he presented at our emergency department. The VA was HM in both eyes. Intraocular pressure (IOP) values were 6 mmHg in the right eye and 4 mmHg in the left eye. Both eyes exhibited deep ocular redness, corneal edema, a few cells and non-granulomatous KP, 2 mm hypopyon, aqueous flare (++), and iris neovascularization (Fig. 2a). The fundus was not visible due to vitreous inflammation. Ultrasound examination revealed bilateral vitreous opacity. According to his history, intravenous ceftazidime and vancomycin plus ofloxacin and steroid drops were administered. However, after 2 days, inflammation remained severe and unchanged.

Based on the uncontrolled inflammatory reaction, invasive treatments, including intracameral or intravitreal injections, and even vitrectomy, were considered. We then learned from the patient’s daughter that he suffered from recurrent moderately painful oral, perineal, and gastrointestinal ulcers for many years, which were relieved by long-term corticosteroid treatment. The daughter and the patient denied ocular complaints in the years prior to his surgery. Due to the possibility of autoimmune uveitis, which might be induced by ocular surgery, invasive operations such as anterior chamber paracentesis or vitreous tap were delayed to avoid further reactive inflammation and possible iatrogenic infection. A panel of immunology tests and an etiological examination were conducted and revealed no significant abnormality. However, based on the typical oral and perineal signs and the ocular attack, a diagnosis of Behcet’s disease was made. Oral prednisone, thalidomide and cyclophosphamide were attempted before any invasive treatment. After 5 days, the inflammation was markedly reduced (Fig. 2b). The inflammation was invisible at his last visit 2 weeks later, after which he returned to his hometown and did not return for further evaluation.

## Discussion

### Differentiation of complications after cataract surgery

Cataract surgery is a relatively safe procedure that typically provides immediate improvement in vision [1]. However, serious complications can occur, including infectious endophthalmitis, toxic anterior segment syndrome (TASS), [2] and sympathetic ophthalmia (SO) [3]. For some patients with systemic immunological conditions, such as Behcet’s disease in this case, uveitis induced by surgery should be expected [4]. In addition, Masquerade syndrome [5] may be considered in elderly patients. With similar signs and symptoms, these conditions could be confusing to surgeons. For infectious endophthalmitis, invasive procedures, such as intracameral and intravitreal injections or vitrectomy, are typically performed. However, for SO and autoimmune uveitis, additional surgery may exacerbate the inflammation.

In this case, the effective treatment history could be misleading. Endophthalmitis might be considered first due to its higher incidence had his past oral and perineal ulcer history not been known. However, there were other clues to guide us: (1) the long interval between surgery and inflammation occurrence, greater than 1 month; (2) the feature of repeated occurrence during a long course of 6 months; (3) the resistance to antibiotic treatment; (4) the very low IOP, 6 mmHg (right) and 4 mmHg (left), argued against acute infection; and (5) the fewer cells and less aqueous flare in anterior chamber compared with severe infection. All of these signs suggest that noninfectious inflammation should be taken into account. TASS could be easily excluded due to the long interval between the surgery and inflammation. Treatments for SO and Behcet’s disease are similar. For Masquerade syndrome, vitreous biopsy and cytological tests could be indicative; however, these procedures are invasive and dangerous to this patient. In our case, given the effectiveness of immunosupressive therapy, Masquerade syndrome could be excluded. A limitation in our case is that only short-term follow-up was possible, and such patients require long-term follow-up to fully evaluate the outcome and potential recurrence.

### Perioperative management of cataract surgery in Behcet’s disease and other uveitis

Studies revealed good vision prognosis for cataract surgery in patients with Behcet’s disease if no active inflammation occurred for 3 months before surgery [6]. In this case, the man did not reveal past ocular history, and no inflammation was observed prior to surgery. Thus, it reminded us that the perioperative management of cataract surgery in such patients should be performed with greater caution. (1) Autoimmune disease history, including oral and skin ulcers and arthralgia, should be included in history-taking. (2) Physical examination and interpretation of auxiliary tests should be carefully performed. Reviewing his perioperative record, we noticed that the preoperative ultrasound revealed bilateral vitreal opacity, and OCT demonstrated epimacular membrane and local serous neuroepithelial detachment in the left eye, which could be indicative of long-term chronic inflammation. Combined with the history, some threatening conditions might be suggested. (3) Uveitis should be inactive for at least 3 months preoperatively, and anti-inflammation therapy should be used preoperatively and continued postoperatively [7]. (4) Even if the awareness of autoimmune disease might have been lacking prior to surgery, these conditions should be taken into routine consideration, just like infectious inflammation, in postoperative follow-up. This would help achieve correct differentiation and treatment of postoperative complications.

## Conclusion

The management of patients with cataract in combination with uveitis is complicated. Once postoperative complications occur, these conditions may be confusing and threatening. Thus, the perioperative management of cataract surgery in such patients should be performed with great care.

## Abbreviations

HM, hand motion; IOL, intraocular lens; IOP, intraocular pressure; KP, keratic precipitates; OCT, optical coherence tomography; SO, sympathetic ophthalmia; TASS, toxic anterior segment syndrome; VA, visual acuity
